# Characterizing the Use of High-Dose Amoxicillin for the Treatment of Bacteremia

**DOI:** 10.3390/pathogens15010054

**Published:** 2026-01-06

**Authors:** Julia Lloyd, Kathleen Lau, Cindy San, Victor Leung, Colin Lee

**Affiliations:** 1 Pharmacy Department, St. Paul’s Hospital, 1081 Burrard Street, Vancouver, BC V6Z 1Y6, Canada; julia.lloyd@cw.bc.ca (J.L.); klau@providencehealth.bc.ca (K.L.); cindy.san@vch.ca (C.S.); 2 Pharmacy Department, BC Children’s Hospital, 4500 Oak Street, Vancouver, BC V6H 3N1, Canada; 3 Faculty of Pharmaceutical Sciences, University of British Columbia, 2405 Wesbrook Mall, Vancouver, BC V6T 1Z3, Canada; 4 Department of Pathology and Laboratory Medicine, University of British Columbia, 2211 Wesbrook Mall, Vancouver, BC V6T 1Z7, Canada; vleung@providencehealth.bc.ca

**Keywords:** high-dose amoxicillin, beta-lactams, bacteremia

## Abstract

Treatment of bacteremia has traditionally consisted of a 7–14-day course of intravenous (IV) antibiotics. Transitioning from IV to oral (PO) antibiotics in uncomplicated cases of Gram-negative and Gram-positive bacteremia is non-inferior to a complete course of IV antibiotics. High-dose oral amoxicillin has been used in practice for treating bacteremia but has limited safety and efficacy data. We conducted a retrospective chart review between June 2022 and June 2024 to characterize the use of high-dose amoxicillin and evaluate its efficacy and safety. A convenient sample size of 100 patients was used. Patients admitted to hospital who received at least one dose of high-dose amoxicillin (1 g PO TID) for the treatment of bacteremia were included. Patients undergoing hemodialysis and patients receiving amoxicillin for other infections were excluded. The average patient was a 60-year-old male (66% male) with a Gram-positive respiratory or skin source bacteremia. The median time to transition to oral amoxicillin was 5 days. The median duration of total treatment was 14 days. Respiratory sources were treated for a shorter duration, whereas skin sources were treated for longer. Readmission to hospital occurred in 28% of cases. The majority of readmissions were unrelated to the original infection, and 92% of patients were cured. There were no observed adverse events, bacteremia relapses, or deaths. In this observational study, transitioning to high-dose oral amoxicillin was primarily used for treatment of uncomplicated respiratory and skin infections with secondary bacteremia. A high rate of clinical success was observed with high-dose PO amoxicillin, with no adverse events reported.

## 1. Introduction

Antibiotic therapy for uncomplicated bacteremia has traditionally consisted of 7–14 days of intravenous (IV) antibiotics. Recent evidence suggests that transitioning from IV to oral (PO) antibiotics in uncomplicated bacteremia is non-inferior to completing a full course of IV therapy. Studies comparing fluoroquinolones (FQs) and sulfamethoxazole-trimethoprim (SXT) have shown promising results [1,2,3]. However, FQs and SXT both carry significant adverse effects (AEs). FQs are associated with tendon rupture, neuropathy, QTc prolongation, and glycemic disturbances [4,5], while SXT is linked to Stevens–Johnson Syndrome (SJS), acute kidney injury, pneumonitis, drug-induced hepatitis, and hyperkalemia. In contrast, oral beta-lactams (OBLs) are generally safe and well tolerated [5,6].

Evidence supporting OBLs in bacteremia remains limited. Mack et al. [7] compared FQs and SXT to OBLs in uncomplicated *Enterobacterales* bacteremia and found no difference in 30-day readmissions, though recurrent urinary tract infections were more common with OBLs. Two studies in uncomplicated streptococcal bacteremia showed no difference in clinical success and treatment failures. Most patients received cephalosporins rather than high-dose amoxicillin in the OBL groups [8,9]. In a retrospective, observational cohort study in Michigan in 2023, Geyer et al. [10] showed that OBLs were as effective as oral FQ and SXT in treating bacteremia from a urinary source.

The optimal OBL agent and dose remain uncertain. Despite the lack of robust evidence guiding the optimal OBL regimen for the treatment of bacteremia, high-dose amoxicillin has been commonly used as the OBL of choice for susceptible pathogens at our institution. Given its good bioavailability and tolerability, evaluating the use of high-dose amoxicillin as a therapy is important. High-dose amoxicillin was chosen over other agents due to its favorable safety profile. The purpose of this study is to characterize the use of high-dose oral amoxicillin at a tertiary care teaching hospital and to determine the safety and efficacy of high-dose amoxicillin in treating bacteremia. The hypothesis of this study is that high-dose amoxicillin is effective and safe for susceptible streptococcal bacteremia. The results of this study will build upon the existing literature regarding the use of OBLs in the treatment of bacteremia and, more specifically, the efficacy and safety of high-dose oral amoxicillin.

## 2. Materials and Methods

### 2.1. Study Design and Setting

This single-arm retrospective observational study without a comparator group chart review was conducted at a single health network, which includes a tertiary care and a community acute care hospital in Vancouver, British Columbia, Canada, from June 2022 to June 2024. This study was approved by the University of British Columbia—Providence Health Care Research Ethics Board.

### 2.2. Study Population

We included hospitalized patients who were 18 years of age or older and who received one or more doses of high-dose amoxicillin (1 g PO TID) for the treatment of bacteremia testing susceptible to ampicillin. We excluded patients who were undergoing renal replacement therapy, had chronic kidney disease (CKD), or received amoxicillin for a different indication unrelated to bacteremia. There was no exclusion based on ward or underlying disease, other than what is mentioned above.

### 2.3. Data Collection and Management

A convenient sample size of 100 was used based on feasibility, and patients were not restricted based on what ward they were admitted to. We collected data until June 2022 to achieve this sample size. Baseline patient characteristics included age, sex, Charlson comorbidity index score, immunocompromised status, Pitt bacteremia score, length of stay, source control status, pregnancy status, concurrent antibiotics, source of bacteremia, and blood culture results. Data was collected using a pre-populated data collection form using Microsoft Excel (Version 2502).

### 2.4. Outcomes

The primary objective was to characterize the use of high-dose amoxicillin in patients with bacteremia. Primary outcomes included median duration of IV antibiotic treatment in days, median duration of high-dose amoxicillin in days, and median duration of total antibiotic treatment in days. Secondary objectives were to evaluate clinical success and safety of high-dose amoxicillin in patients with bacteremia. Secondary outcomes included the proportion of patients achieving clinical resolution (defined as a reduction in signs or symptoms at discharge or end of treatment), readmission at 30 days, relapse with same bacteremia at 30 days, mortality at 30 days, patients requiring a change in oral amoxicillin therapy due to any adverse effects, and ADRs grade 3 or above reported as per the Common Terminology Criteria for Adverse Events. Subgroup analysis based on the source of bacteremia and time to oral amoxicillin transition (early vs. late switch) were also explored. An early switch was defined as transitioning to oral amoxicillin after 5 or less days of intravenous therapy, and a late switch was defined as transitioning to oral amoxicillin after 5 days of intravenous antibiotic therapy.

### 2.5. Statistical Analysis

Baseline characteristics and outcomes were summarized using means (standard deviations), medians (interquartile ranges), and percentages where appropriate. Subgroup analysis of early vs. late switch for the primary outcome of total duration of antibiotics was assessed with a Mann–Whitney U test as the data was considered skewed when plotted on a histogram. This was performed done using Microsoft Excel (Version 2502) and considered significant if *p* < 0.05.

## 3. Results

**Figure A1:**
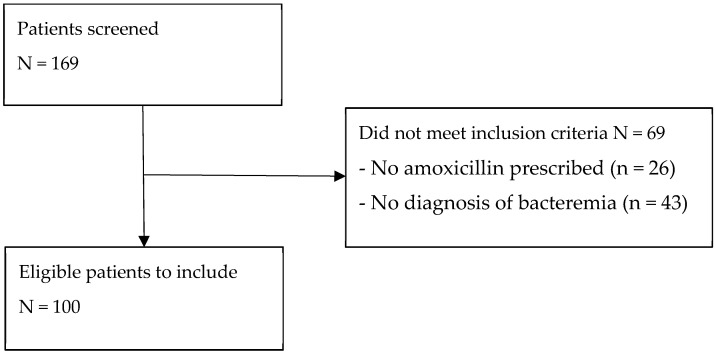
Flow diagram of included patients.

A total of 169 patients were screened based on inclusion and exclusion criteria to reach a convenient sample size of 100 patients (Figure A1). The average patient was a 60-year-old male (66% male) with a Charlson comorbidity index of 3.4 and Pitt bacteremia score of 1.3 (Table 1). The most common source of infection was skin and soft tissue (42%), followed by respiratory (33%) (Table 1). The majority of infections were considered uncomplicated as most patients were not critically ill at admission, and there were few deep-seated infections and no cases of persistent bacteremia. The most common bacteria identified from blood culture results was beta-hemolytic streptococci (46%), followed by *Streptococcus pneumoniae* (31%) (Table 1). 

**Figure A2:**
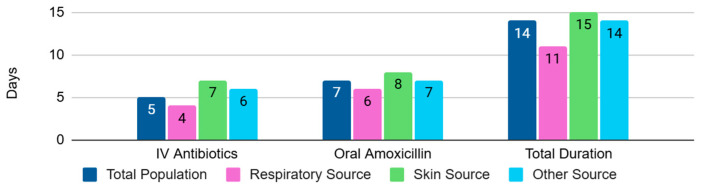
Median duration of antibiotic treatment.

**Figure A3:**
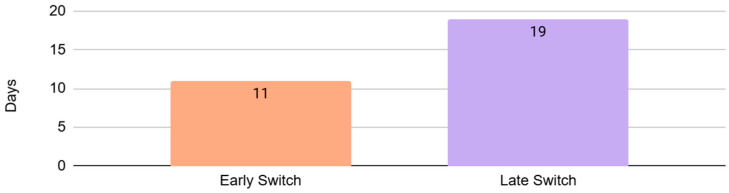
Mean duration of total antibiotic treatment in early vs. late switch.

The median duration of IV antibiotics was 5 days for the total population (4 and 7 days for respiratory source and skin and soft tissue infections, respectively). The median duration of high-dose amoxicillin was 7 days for the total population (6 and 8 days for the subgroups of respiratory source and skin and soft tissue infections, respectively). The median duration of total antibiotics was 14 days for the total population (11 days for the respiratory infections and 15 days for the skin and soft tissue infections) (Figure A2). The median duration of total antibiotics in the early and late switch subgroup was 11 days and 19 days, respectively, this difference being statistically significant, with a *p*-value of <0.001, with Z = 4.33 and U = 607.5 (Figure A3). Only 11% of patients had a total treatment duration of 7 days or less. Of these patients, 82% had a bacteremia from a respiratory source, with the majority of these cases attributed to *Streptococcus pneumoniae*. It should be noted that most patients received at least one dose of IV antibiotics prior to oral amoxicillin, with agents varying between patients.

Clinical resolution was achieved in 92% of included patients. The rates of clinical resolution were similar among early and late switch patients with 93% and 91%, respectively. A readmission rate of 28% was observed at 30 days, with a readmission rate of 23% and 31% in early vs. late switch, respectively. No patients had a relapse of the same bacteremia at 30 days as no patients were readmitted with the same pathology, no patients died at 30 days, and no patients reported adverse drug events requiring a change in amoxicillin therapy (Table 2). 

## 4. Discussion

This retrospective observational study found that high-dose amoxicillin was primarily for the treatment of uncomplicated respiratory and skin and soft tissue infections with secondary bacteremia from streptococcal species. The median time to transition to oral amoxicillin was 5 days, and the median duration of total treatment was 14 days. There were high rates of clinical resolution (92%) and low rates of readmission (28%), with most of these readmissions unrelated to infection, and no cases of relapse or reported adverse events. 

This study differs from the previously published literature where PO transitions to OBLs were variable in terms of agent, dosing, and frequency. Most transitions were to amoxicillin–clavulanate, with a median duration of 14 days of treatment. There is a lack of data looking specifically at high-dose amoxicillin as the oral transition option for the treatment of bacteremia. Although there was no direct comparator group in this study, the rates of clinical resolution observed were similar to previously published studies where clinical cure was observed to be 94% [8,11]. Rates of adverse events and readmission were lower in our study compared with the previous literature, where adverse reaction rates were 36% and readmission rates were 7% [8,11]. Most readmissions found in this study were not related to the chief complaint of the original bacteremia but instead due to non-infectious reasons, such as overdose or withdrawal. Our study differs from previous studies, as they looked at various OBLs with various dosing regimens as an oral transition therapy but not specifically at high-dose amoxicillin [8,11]. High-dose amoxicillin was chosen for the reason highlighted in the study by De Velde and colleagues that target attainment can be achieved for higher MICs of 1 mg/L, compared with lower doses [12].

There were some limitations with this study. The majority of amoxicillin prescriptions were dispensed in the outpatient setting, and adherence and safety after discharge from hospital was not captured. Other limitations included having no control group and the retrospective chart review with small sample size. Nevertheless, our study still showed a high rate of clinical resolution which was similar to the previously published literature, which had similar study protocols, varying mostly by the transition to variable OBLs in comparison to only high-dose amoxicillin. The study is at risk of selection bias as the population being transitioned to amoxicillin at this site may differ from the general population as many patients were likely to have a patient-initiated discharge, and therefore prescribers are more likely to transition to a well-tolerated oral antibiotic to treat the bacteremia, rather than a prolonged course of IV antibiotics that require a hospital stay. The majority of bacteremias were uncomplicated streptococcal bacteremias and may not be generalizable to bacteremias due to Gram-negative organisms. Lastly, the total duration of therapy was longer than we expected (median 14 days), making it difficult to extrapolate the efficacy of amoxicillin with a shorter treatment duration which is now recommended for certain cases of bacteremias. This is highlighted in the review article by Davar et al., where they cite more than 120 randomized controlled trials that have established that shorter durations are equally effective for many infections, including some bacteremias [13]. As well, a recently published BALANCE study confirmed that 7 days of antibiotic treatment was non-inferior to 14 days among hospitalized patients with bloodstream infection. Despite being a well-designed RCT with a large sample size, there were only a small number of Gram-positive bacteremia included and no subgroup analysis of the outcomes of those who were transitioned to oral beta-lactam antibiotics. Our study specifically focused on the subgroup of Gram-positive bacteremia and the outcomes with completing treatment with a defined dose of oral amoxicillin [14].

Future research should include comparator groups such as other oral beta-lactams or FQs and SXT, and even standard doses of amoxicillin as a transition therapy. It would also be important to address the optimal duration of therapy with future studies.

Transition to high-dose amoxicillin appears to be safe and efficacious in the treatment of ampicillin-susceptible streptococcal bacteremia of respiratory or skin/soft tissue origin. This study should not be extrapolated to Gram-negative bacteremias, complicated infections, or conditions such as endocarditis or meningitis until further research is performed. This practice should be considered to reduce risks associated with prolonged IV antibiotics and other oral options which are associated with higher rates of adverse effects.

## Figures and Tables

**Table 1:** Baseline characteristics (N = 100).

Age (years)—mean (SD)	60.1 (19.0)
Sex (female)—no. (%)	34 (34)
Charlson comorbidity index—mean (SD)	3.4 (2.8)
Immunocompromised—no. (%) ^1^	17 (17)
Pitt bacteremia score—mean (SD)	1.3 (1.9)
Length of stay (days)—median (IQR)	9 (5,15)
Source control achieved—no. (%) ^2^	16 (100)
Pregnancy—no. (%)	1 (1)
Receiving another antibiotic—no. (%)	20 (20)
**Source of bacteremia—no. (%)**	
Skin	42 (42)
Respiratory	33 (33)
Genitourinary	15 (15)
ENT	4 (4)
GI	2 (2)
No clear source	2 (2)
**Blood culture results—no. (%)**	
Beta-hemolytic streptococci ^3^	46 (46)
Streptococcus pneumoniae	31 (31)
Escherichia coli	8 (8)
Mixed/other ^4^	8 (8)
Haemophilus influenzae	4 (4)
Enterococcus faecalis	3 (3)

^1^ Immunocompromised as defined in protocol (current chemotherapy, immunosuppressive therapy, chronic corticosteroid use (>30 days), uncontrolled HIV/AIDS). ^2^ Source control for infections that required source control (*n* = 16). ^3^ Group A Streptococcus (29%), Group B Streptococcus (8%), Group C Streptococcus (1%), Group G Streptococcus (8%). ^4^ Streptococcus Gondii, Actinobacillus Ureae, Streptococcus Parasanguinis, Proteus Mirabilis.

**Table 2:** Secondary and safety outcomes—no. (%).

	Total Population (N = 100)	Early Switch (N = 43)	Late Switch (N = 57)
Clinical Resolution	92 (92)	40 (93)	52 (91)
30-Day Readmission	28 (28)	10 (23)	18 (31)
30-Day Relapse	0 (0)	0 (0)	0 (0)
Mortality	0 (0)	0 (0)	0 (0)
Adverse Events	0 (0)	0 (0)	0 (0)

## Data Availability

The data generated during the current study are available from the corresponding author upon request.

## Abbreviations

The following abbreviations are used in this manuscript:

IV	Intravenous
PO	Oral
FQ	Fluoroquinolones
SXT	Sulfamethoxazole-trimethoprim
SJS	Stevens–Johnson syndrome
OBL	Oral beta-lactam
CKD	Chronic kidney disease
